# The neuroimaging magnitude of pediatric brain atrophy in northern Tanzania

**DOI:** 10.11604/pamj.2020.36.25.22515

**Published:** 2020-05-21

**Authors:** Richard Erasto Sungura, John Martin Spitsbergen, Emmanuel Abraham Mpolya, Elingarami Sauli, John-Mary Vianney

**Affiliations:** 1 Department of Health and Biomedical Sciences, School of Life Science and Bioengineering, Nelson Mandela-African Institution of Science and Technology, Arusha, Tanzania; 2 Department of Biological Sciences, Western Michigan University, Michigan, United State of America

**Keywords:** Brain atrophy, brain volume, evans index, neuroimaging

## Abstract

**Introduction:**

The loss of parenchymal brain volume per normative age comparison is a distinctive feature of brain atrophy. While the condition is the most prevalent to elderly, it has also been observed in pediatric ages. Various causes such as trauma, infection, and malnutrition have been reported to trigger the loss of brain tissues volume. Despite this literature based knowledge of risk factors, the magnitude of brain atrophy in pediatric age group is scantly addressed in most developing countries including Tanzania. The current study aims to understand the magnitude of brain atrophy in children residing in Northern Zone, Tanzania.

**Methods:**

A cross-sectional hospital survey was performed in which 455 children who were presented with various brain pathologies from the year 2013 to 2019 and whose brains examined by Computerized tomography (CT)-Scanners were recruited in the study. The brain statuses were examined using three linear radiological methods including the measure of sulcal-width, Evans index, and lateral ventricular body width.

**Results:**

Results showed a significant number of atrophied brains among children in Northern Tanzania and that the condition was observed to have a 1:1 male to female ratio. The prevalence of pediatric brain atrophy was found to be 16.04%.

**Conclusion:**

The cortical subtype of brain atrophy presented as the most prevalent type of brain volume loss. The findings of this study suggest existence of considerable trends of brain atrophy in children which need special attention and mitigation plans.

## Introduction

Brain atrophy being unusual reduction in brain volume than expected in the normal development and involution of brain at a particular age, is an important clinical condition in the current and future perspectives of neuroscience and related disciplines in studying neuro-cognitive functions and anatomical structures of the human brain. When fully developed, the atrophied brain manifests with reduction in parenchymal volume with the corresponding enlargement of sulcal spaces, basal cisterns and ventricles which are occupied by increased cerebrospinal fluid volume as a result of ex-vacuo response [1]. Though the process of brain volume loss is commonly found in elderly, there is a number of evidences on the similar observation in childhood [2].There are also various pathological events that have been closely associated with loss of brain tissues and affect the normal anticipated course of brain tissue proliferation in childhood. Among causes of brain atrophy involve birth asphyxia [3], inborn errors of metabolism [4], trauma [5], infection [6], malnutrition [7], cytotoxic drugs and radiation injuries [8]. In developing countries including Tanzania, the magnitude of brain atrophy in children remains uncertain in most of scientific literatures as not much has been studied and published. Most studies cover the aspects of brain atrophy as part of neurodegenerative changes in elderly such as Alzheimer´s disease, multiple sclerosis, Cerebral autosomal arteriopathy with subcortical infarcts and leukoencephalopathy (CADASIL) syndrome [9] and other white matter changes [10]. Prevalence of brain atrophy therefore has been frequently covered as an entity among other disease conditions such as liver cirrhosis [11] and alcoholism [12]. Such conditions are rare in childhood, therefore makes brain atrophy to be more construed as a disease of old age [13]. In Tanzania brain atrophy in childhood is becoming a common condition in neuro-imaging settings. However, no study has addressed this condition in childhood and hence its prevalence in the country remains unknown. Studies have shown that, to a significant extent pediatric brain atrophy is associated with central nervous system infection such as malaria and HIV-encephalopathy [6], trauma and birth related brain injury to mention a few [14]. These pathological processes are also common in the country, thus why the current study seeks to understand the status quo of the brain atrophy in children by determining its prevalence so as to set baseline information for the magnitude of brain atrophy and its burden in Tanzania, specifically Northern Zone of the country.

## Methods

**Subjects and image acquisition:** we studied 455 young patients who were presented in radiology departments of health facilities in the Northern Tanzania and performed brain CT scan examinations between the years 2013 to 2019. All patients underwent CT scan brain using primary axial cuts with slice thickness of 5mm and increment of 2mm. All the images were taken along the standard radiological baseline.

**CT scan image analysis:** brain CT scan images were examined using the three known radiological linear methods to determine the presence or absence of brain atrophy. The similar measurements were used to differentiate the subtypes of brain atrophy including global atrophy, central atrophy, cortical atrophy, focal atrophy and hemi-atrophy. While the Evans index (EI) and lateral ventricle body width (VW) measure the central type of brain atrophy [15], the cortical sulcal width (SW) measure the cortical or peripheral type of brain atrophy [16]. The atrophy in one lobe or part of a lobe it is categorized as focal brain atrophy [17] and a loss of volume in one and whole hemisphere is referred as brain hemi-atrophy [18]. Global atrophy involves both cortical and central atrophy bilaterally resulting into delocalized or whole brain volume reduction in a symmetrical fashion [19].

**Statistical analysis:** data were analyzed by R-statistical package where mean and p-values for age and gender association, and brain ventricular size and sulcal width were obtained. The percentage of subjects with brain atrophy or normal brain was calculated.

## Results

**Figure 1:**
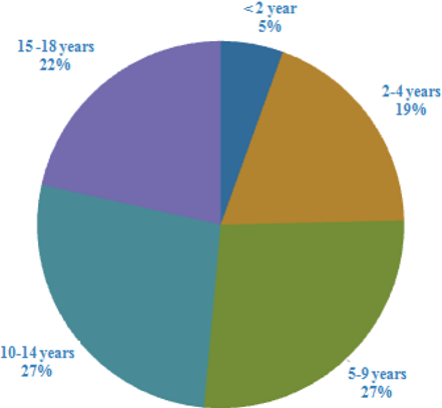
Demographic characteristics of children in cross section survey

**Figure 2:**
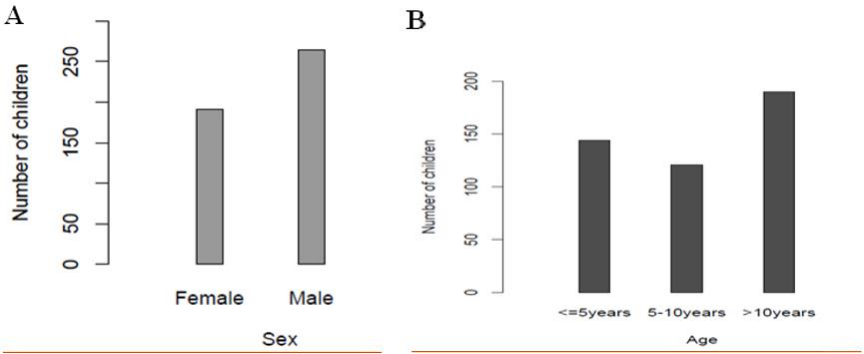
Gender and age distribution of children in the study: (A) the distribution showing more male children than female in the study population were taken in healthy facilities for brain CT scan examinations; (B) children population in clusters of 5 years intervals showing the over 10 years being the dominant population followed by the under 5 years

**Demography:** the demographic characteristics showed that the predominant population involved children above 10 years of age, while the under five constituted a relatively lower population (Figure 1, Figure 2A). In this study males were 264 while females were 191 equals to 58% and 42% respectively, indicating that the male children who attended hospitals for CT scans were more than female children. Therefore, the mean age of all participants was 9.25 (±5.24) years (Figure 2B).

**Comparison of radiological measurements:** the quantitative evaluation of children brains using the three linear radiological measurements showed that sulcal width (SW) and lateral ventricular width (VW) had P-value <0.05 suggesting significant association with age while the Evans Index (EI) had P-value of 0.431 indicating less significant age association (Table 1). When gender association was considered, the three radiological linear measurements for all THREE measurements SW, VW and EI had insignificant gender association with p-vale greater than 0.05 (Table 1). The SW and EI have shown variation presented by high standard deviation in both males and females (data not shown).

**Table 1:** Brain dimensional measurements for all children, and correlations of dimensions with age

	n	Mean(±SD)	Age and dimension correlation (rho)	Correlation test (p-value)	F test
Sulcal width	357	1.94(±0.70)	-0.19	0.0004	11.009***
Ventricular width	346	24.99(±6.98)	-0.23	0.001	6.4977*
Evans	346	0.34(±1.38)	0.04	0.431	17.146ns

Note: statistically significant at

* p < 0.05

**p < 0.01

*** p< 0.001; ns = not significant

**Figure 3:**
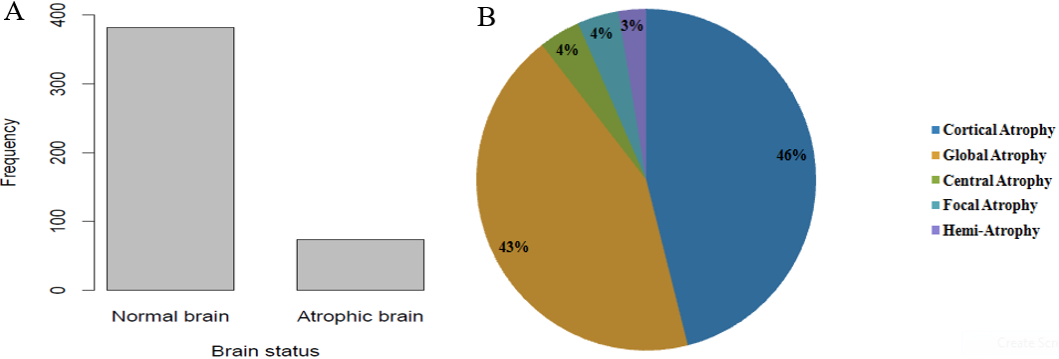
The magnitude of brain atrophy and its sub-types as per three known linear radiologic methods in Northern Tanzania: (A) the two groups represents (i) children who measured sulcal width < 2.5mm, ventricular width < 30mm and evans index < 0.3 as children with normal brain volume; The minor group involved children with higher values of sulcal width, ventricular width and Evans indices as atrophied brain cases; (B) Morphological characterization of the patterns of brain volume loss is presented in total of five forms with their percentage distribution in the studied population

**The status of brains volume:** in the total of 455 children who were reviewed, 73 children equals 16.04% presented with brain volume reduction, that is brain atrophy (Figure 3A). The remaining 382 equals to 83.96% children showed normal brain volume or had other brain pathologies not related to brain volume loss.

**Figure 4:**
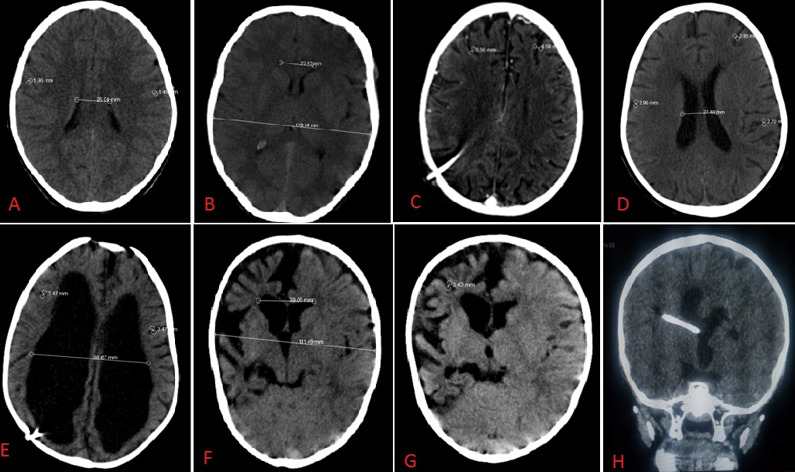
Varying brain volumes, normal versus atrophied brain: (A) normal pediatric brain volume showing sulcal width < 2.5mm and Lateral ventricular width < 30mm; (B) normal pediatric brain volume whose distance of the anterior horns of lateral ventricle and the widest diameter gives the Evans Index of 0.2 (normal is < 0.3); (C) global brain atrophy-after long standing hydrocephalus which was shunted then exposing prominent salcal spaces; (D) cortical brain atrophy showing prominent sulci but normal lateral ventricle (< 30mm); (E) global brain atrophy, a post intraventricular hydrocephalus showing prominent lateral ventricular width and sulcal space; a VP shunt is noted at right parieto-occipital junction; (F) brain hemiatrophy showing severe loss of volume in the right hemisphere: Evans index is 0.34; (G) brain hemiatrophy showing prominent sulcal spaces in the right hemisphere (SW > 0.25mm); (H) coronal image of right brain hemiatrophy with ipsilateral congenital megaloncephaly, VP shunt in ventricle

**The patterns of brain volume loss:** of the Brain atrophy cases presented, about five patterns of brain atrophy were observed. These included cortical atrophy, global atrophy, central, focal and hemiatrophy with the percentage of 46%, 43%, 4%, 4% and 3% respectively (Figure 3B). Some varying patterns of typical brain volume loss are shown as they appear on CT scan images (Figure 4). Further scrutiny of atrophic cases showed the variegated trends in levels of vulnerability whereas children below 5 years of age were the most vulnerable compared to children above 10 years of age (data not shown).

## Discussion

The purpose of this study was to determine the magnitude of brain atrophy as seen among children in Northern Tanzania. In this study, we show that the brain atrophy occurs mostly in children less than five years old compared to children of higher age groups in the population. The under-five children are likely to be affected more than the older children by this condition in connection to their immature respiratory and immune system resulting into higher propensity to both central nervous system and respiratory tract infection [20], the conditions that may results into brain cells damage [21]. The children above-five show low vulnerability with age advancement due to a possibility that brain atrophy to a large extent is incompatible with life in childhood hence atrophied brain could not be carried forward to late childhood and adolescence due to mortality as per the study by Kulak *et al.*, 2003 [22] who demonstrated that brain atrophy was a poor prognostic indicator for children with epilepsy. Further studies suggest that the degree of brain tissues damage is directly related to poor functional recovery [23]. Therefore, mortality is a considerable explanation for low trend of brain atrophy in late childhood.

Gender distribution of brain atrophy for the population who participated in the current study seem to be equal for both male and female children, this implies insignificant correlation of brain atrophy with gender. This observation is possibly due to overall equal propensity to the risk factors of brain atrophy during childhood compared to adolescence and adulthood in which traumatic brain injury is a common risk factor with higher male to female ratio [24]; furthermore, the hormonal mediated secondary sexual characteristic at studied age range has insignificant impact on brain differentiation influenced by gonadal hormones. The gonadal hormones namely estrogen, progesterone and testosterone [25], have influence on brain development but more importantly estrogen has shown neuro-protective role in adult females as evidenced by rapid brain atrophy and demyelination in post-menopausal period after estrogen withdrawal [26]. The estrogen role is thought to be mediated by an interaction with insulin like growth factor (lGF-1) [27]. Progesterone has concurrently been reported to facilitate repair of brain cells after traumatic brain injury [28]. In a nutshell, gonadal hormones´ significance in brain growth and maintenance exists in varied mechanisms. The Evans index(EI) insignificant correlation in pediatric brain atrophy, can be explained by the fact that Evans index nominator is measured through the frontal horns of the ventricle while myelination (maturation of white matter) is known to start from occipital toward frontal lobes during childhood and hence less change in Evans index values is anticipated [29]. Therefore, the use of multi-parametric assessment of brain volume improves depiction power of brain atrophy in varying ages. The variations involving sulcal width and Evans index with high standard deviations can be due to presence of transitional states that affect structural shape of skull and brain volume. For instance, hospitalized patients and especially children if are over hydrated with I.V fluids they tend to have temporary increase in body fluids [30] and when dehydrated after conditions like diarrheal diseases children tend to have reduced intracranial fluid, overlapping sutures and sunken fontanels hence resulting into variation in the measured dimensions irrespective of similarities in age factor [31]. The similar condition might have happened in the current study, although, these parameters were beyond the scope of this study. The cortical type of brain atrophy was more prevalent type of brain atrophy followed by the global brain atrophy. This observation could be accounted by the possibility that the determinants of brain atrophy in these two categories are likely to be the results of systemic pathological processes like drug mediated brain injury and malnutrition such as marasmic-kwashiorkor [32]; the vast majority of systemic conditions are likely to cause generalized and symmetrical cerebral volume loss with surprisingly no significant change in brain stem and cerebellum. Furthermore, the cortical atrophy is mostly the outcome of volume loss in grey-matter than the white matter [33]. Under normal circumstances studies have shown evidence of more brain perfusion in grey-matter than white matter to meet metabolic demands, hence this is in alignment with the findings of this study that systemic determinants of brain atrophy tend to have predilection to the brain tissues with high metabolic demand normally, these brain parts are characterized by high calculated perfusion values just as the way grey matter shows higher average enhancement value than white matter in CT perfusion study [34]. Therefore, the patterns of brain atrophy may reflect the nature of the brain injury determinants. Hemi-atrophy seems to be rare according to this study and likewise in the overall global statistics [35]. The reason for this could be explained by the high possibility that the determinants of brain hemi-atrophy are more responsible for the condition during intra-uterine life than post natal life [36]. From the light of literature, among other reasons for brain hemi-atrophy is an ischemic event or vascular occlusion during pre-natal period that results into lateralized or localized brain hemispheric volume loss with sparing of the other hemisphere and hence asymmetrical brain development [37]. From this back ground, asymmetrical skull shapes are common findings in children with brain hemi-atrophy.

Meanwhile, the central brain atrophy can be a result of a previous pathological condition. In the current study population, the central brain atrophy seems to be the result from a previous hydrocephalus with white matter change due to seepage of cerebro-spinal fluid before treatment intervention by ventriculo-peritoneal shunt is instituted [38], whereas in different population, multiple sclerosis was reported to be involved in causing a central brain atrophy [23], suggesting that the means of prevention, diagnosis, and treatment of central brain atrophy may be diverse. In this and other studies, cases of white matter demyelinative disease are rare in childhood compared to adult; hence purely central type of brain atrophy is not a prevalent form of volume loss compared to cortical and global atrophy. While the brain atrophy is construed as a common condition in elderly [39], this study has presented a 16.4% of brain atrophy prevalence in children contrary to the only available local data from the study by Muola D, 2017 accessed through the Muhimbili University of Health and Allied Sciences´ repository in Tanzania. In this local study, brain atrophy prevalence of about 13% was reported among adult patients with white matter diseases [40]. Other studies suggest a very close relation between brain atrophy and white matter diseases in senile age [41]. The brain atrophy prevalence of 13% in adults is equivocal for the age and is not in tandem with the concurrent presence of white matter diseases; such a finding is likely to have been eluded by technical issues related to lack of reproducible quantitative tools for measuring and diagnosing brain atrophy [42]. The study done in Ibadan West Africa presented the brain atrophy prevalence of 10.6% in children [43], which is close to the results of our study by virtual of age correlation. Therefore age related studies focusing on the quantitative neuro-investigations are the way to address the true burden of brain atrophy in children and other age groups as brain volumes varies significantly with age [44]. Most adults in fifth decade of life have brain atrophy of senility hence they cannot be regarded in the same manner as children [45]. Higher magnitude of brain atrophy is anticipated and may be accelerated by other pathologies such as diabetes [46], stroke, alcoholic encephalopathy and liver cirrhosis among other conditions which are seldom in childhood [47]. Even though, multiple co-morbidities explain why brain atrophy appears to be the disease of elderly, more cases detection can be achieved with the optimal use of quantitative techniques in children neuro-imaging.

Vulnerability levels seem to be closely associated with age such that the under-five years were more affected by brain atrophy compared with children between 4 and 10 years of age. The children above 10 years were least affected by this condition. This could be due to more risk factors such as low immunity to infection, incidence of trauma [23] and birth related injuries at this early age [48]. A study by Zimmern *et al.* (1979) showed various brain areas of infarcts in some children who were abused, all those children in the study developed brain atrophy [49], Similarly, Pinto *et al.* (2012) showed the plasticity of pediatric skull contributes to specific patterns of injuries of young children brains [50] and therefore, the degree of the brain trophy depends on the severity of the brain injury [51]. Life incompatibility with brain atrophy remains to be among concrete reasons why the prevalence of the pathology is less above five years old as most children with severe brain atrophy or injury might have not proceeded further to early adolescence with irreversible forms of this condition [52]. Therefore, an orderly pattern of brain atrophy magnitudes in childhood goes in tandem with distribution of age related risk factors for brain tissues damage in the course developmental mile stones children growth.

## Conclusion

Brain atrophy is paradoxically becoming common in childhood, pressing for the needful adoption and use of quantitative neuro-imaging techniques to attain correct diagnosis and classification of this condition. There is unprecedented significant number of children with childhood brain atrophy such that the current prevalence of pediatric brain atrophy in Northern Tanzania is 16.04% bearing a1:1 male to female ratio. Cerebral Cortical Atrophy is the most prevalent type of brain atrophy in childhood while Brain Hemi-Atrophy is the rarest form of brain atrophy sub-type in the studied population. The under-five children are more vulnerable in developing brain atrophy; hence putting in place programs for early neuro-imaging investigations may revolutionize the current clinical practice in developing countries by virtual of early depiction, grading and mitigation of disease progression into severe forms. The preferential selection of imaging modalities with non-ionizing radiation such as trans-cranial sonography and Magnetic Resonance Imaging stands to be the best consideration in young pediatric age groups.

### What is known about this topic

- The brain atrophy is a condition found most commonly in elderly and may however be found in children especially related to malnutrition, head injury and central nervous system infection;
- Most causes of brain atrophy are currently irreversible.

### What this study adds

- Pediatric brain atrophy is not uncommon. In the Northern Tanzania a prevalence of 16.04% has been depicted;
- Vulnerability to brain atrophy has been shown to be higher in children under-five years of age;
- Among the subtypes of brain atrophy, the cerebral cortical pattern of atrophy is most prevalent in the studied population.

## Competing interests

The authors declare no competing of interests.
